# The Ottoleungui Method of Anæsthetizing Sensitive Dentine

**Published:** 1888-12

**Authors:** Levitt E. Custer

**Affiliations:** Dayton, O.


### THE DENTAL REGISTER.

VOL. XLII.] DECEMBER, 1888. [No. 12.

### COMMUNICATIONS.

### The Ottoleungui Method of Anæsthetizing Sensitive Dentine.

BY LEVITT E. CUSTER, B.S., D.D.S., DAYTON, O.

[Read before the Ohio State Dental Society, Cincinnati, October 16, 1888.]

In a recent number of the *Archives of Dentistry* there appeared the names of several of our leading brethren and their favorite methods and medicaments for obtunding sensitive dentine. The agents used by them would complete a list commencing with mechanical means, followed by dehydrants, coagulators, sedatives, local anæsthetics and closing up with a long roll of patent medicines. Alcohol headed the list of dehydrants, while carbolic acid was the proud exponent of coagulation; at one extreme would be found hot air, and at the other rhigolene; on one hand would be found sharp burs and a Bonwill engine, and on the other "Oliver's Pain Obtunder" and Christian Science, equal parts. Every operator's method was the best, and he was always successful; but recently the attention of the profession has been called to a new method of obtunding sensitive dentine. Dr. B. A. R. Ottoleungui, a young practitioner, baffled in his attempts to satisfactorily obtund dentine by ordinary methods, has, according to his own statements, followed a course of logical reasoning to a triumphant end. Out of over one hundred operations he records no failure, and Atkinson says it is our own fault if we have not tried a method of such statistics.

The author of the method uses the term "anæsthetize" in preference to the old term "obtund," on account of the complete freedom from pain brought about; and since he claims absolute success in every case, let us examine the theory and the action of the different agents employed.

The object is first to withdraw the water from the dentinal canaliculi, leaving a beaded fibril which will not completely fill the tubule, then anæsthetize the fibril. For the former purpose he uses hot air, and for the latter sulphuric ether. He proceeds in the following manner, which I give in his own words: "Isolate the tooth, and one or two others on each side of it, with rubber dam. After using bibulous paper, apply dry heat, not too hot, with the chip blower. These blasts should be continued until the whole tooth becomes whitened, which is sufficient evidence of dehydration. Having done this, the next step is to anæsthetize the fiber. To do this, use the purest ether, thrown on with a continuous spray apparatus." This is continued until the patient is quiescent.

In this method we see a combination of two heretofore well known principle—complete dessication followed by reduction of temperature.

The histological structure of dentine being a tube inclosing a beaded thread of nerve tissue (if we may call it nerve tissue) and the inter-beaded spaces filled with a fluid which we suppose to be water, we see the latter to be essential to perfect sensitiveness of dentine in a two-fold manner; but which surrounds and fills the interbeaded spaces of the dentinal fibril, and that which is a constituent of the fibril itself. The water surrounding the fibril is essential in preserving an equilibrium of pressure, and acts in the same manner in the endolymph of the labyrinth of the ear. One theory of the sensitiveness of dentine is that its tubuli contain only a fluid which alone transmits pressure and sensation to the pulp; but a better theory is, that it, containing this beaded fibril, the fluid is not a continuous column, and in that manner, being enclosed in unyielding walls, presses upon the fibril itself—not the pulp. So if this be true, you will readily comprehend the external relation of the water to the fibril, which, if withdrawn, will reduce the sensitiveness.

In regard to the relation of water as a constituent of nerve tissue or the dentinal fibril, as one of the conditions for the perfect performance of nerve function, there must be moisture; hence, the effect of dehydration and hence its application and reputation as an obtundent for sensitive dentine. By the extraction of water from the tubuli and a portion from the fibril itself, by chloride of zinc, alcohol, hot air, or by whatever means employed, the above condition is stricken out, and sensation cannot be conveyed. The reader never had success in drawing out the water until experimenting according to the first step of the Ottoleungui method, when he found that he had so thoroughly dessicated the dentine and its delicate contents, and it was so nearly devoid of sensibility that the operation was often painlessly completed without applying the ether spray. Complete dessication, by whatever means, to my mind is the most powerful factor in obtunding sensitive dentine, and when used in conjunction with ether spray, is the more effective of the two. I have had very little success at any time with those agents which coagulate albumen.

The second step of the process is simply anæsthesia of the dentinal fibril, which is produced by reducing the temperature by means of the ether spray. Here is a new application of an old method of local anæsthesia—the use of the ether spray for sensitive dentine; as, not many years ago, when the extraction of teeth was of more common occurrence, ether was sprayed upon the soft tissues surrounding the offending member and the operation painlessly performed. So to-day this same agent has been revived and is employed in lessening the pain incident to preparing cavities, filling and saving teeth. As a condition also for the perfect performance of nerve function, every animal organization seems to demand a certain temperature, therefore if that temperature be lowered, there will be a reduction of sensibility in a proportion not unlike the number of degrees. The first sensation is felt and is painful according to the degrees from the normal temperature. If the agent be continued, the nerve tissue itself becomes reduced in temperature and its conveying power is lessened in proportion to its departure from normality—hence the application of the ether spray as a local anæsthetic. Upon cutaneous tissue the benumbing power is aided by withdrawal of blood and nutrition from the parts, but when applied to the dentinal fibril it is dependent entirely upon the refrigerant quality.

The first and unfailing querry arises as to the effect of such extremes upon the pulp. In drying out the dentine it is not understood that the tooth is to be heated at all. Dr. Ottoleungui says the blasts may be so regulated that the whole tooth will not become hot and at the same time the moisture will have been vaporized. My own experience is, that as dentine is a poor conductor, by lengthening the intervals and applying sharp, quick, hot blasts, thorough dessication might be accomplished without the heat reaching the pulp. Therefore it is not necessary that there be pulp injury from the dessicating process.

The only part admitting of doubt is the reduction of temperature. Now it is not intended that this process be continued until icicles hang from the tooth, but simply to that point where there is a cessation of pain, which, fortunately is many degrees from the freezing point. The pulp is not frozen at all; it, and its nerve endings have simply been reduced in temperature until, according to a former argument, they cannot receive or transmit pain to the censorium. Dr. Ottoleungui thinks the fibrilæ alone are anæsthetized, but I am of the opinion, from the very limited duration of anæsthesia, that the pulp also largely shares the benumbing influence. I find as soon as warmth returns by ingress of warm blood and by conduction, sensation is also restored, proving that actual freezing had not taken place, and hence no injury of pulp tissue. Doubtless if this operation were often repeated a chronic lesion of the pulp would set in which would lead to its destruction; but for a single or a second operation I can see no objection. The author says he has seen no outward ill results so far. At this juncture Dr. R. L. Cochran, who has secretly used the ether spray for sensitive dentine for a long time, says: "In twenty-three years I never saw a dead pulp or any ill effects, except sometimes sore mouths, from the ether spray." The latter we would understand to have been due to the ether coming in contact with the mucous membrane, which may be attributed to carelessness rather than necessity. In my own limited experience I have seen no bad effects so far.

The application of cold as an anæsthetic to an organ containing, as it does, nerve endings, whose mission is to cry out against such extremes, may be regarded as heroic treatment; but heroism is what cuts through and burrs out Atkinson's diseased apices of roots; heroism is what surgically treats live pulps; yes, heroism is what implants teeth. Often in our practice it is necessary to become quite severe that thoroughness and success may result. Ottoleungui found the application of the ether spray to be painful in about forty per cent. of cases, but in my hands it has been more or less painful in all, but of such a character that the patient would always prefer it to withstanding the pain of cutting without its use. The pain is most severe at the beginning, but it gradually lessens until at the end of from one to one and a half minutes it has entirely subsided.

As to the effectiveness of this method of obtunding sensitive dentine, I would say that, while it is not the ideal method, after having tried nearly every one known, in my hands it is the only one that will produce complete anæsthesia of dentine. This last stage of completeness may possibly be due to slight general anæsthesia by inhalation of the vapor; at any rate, it is effective; but, whether or not by blundering manipulation, I have not been able to carry the anæsthetic stage long enough for completing a lengthy operation.

The Ottoleungui method of anæsthetizing sensitive dentine by virtue of,at least,the one moment of complete anæsthesia which it produced, is valuable, and for that one long desired virtue is to be recognized as one of the best methods for obtunding sensitive dentine and allaying the terror so commonly associated with the term dentist.

DISCUSSION.

*Dr Berry*—Common air is a very good anæsthetic. For the knowledge of this we are indebted to the important discovery of Dr. Bonwill. If the patient can be induced to breathe rapidly for one minute he will be so anæsthetized that a sensitive cavity in a tooth may be excavated, while the rapid breathing is kept up, without pain. The operation will be felt but usually without pain.

*Dr. H. A. Smith*—This subject of treatment of sensitive dentine is an interesting one. There are one or two points I would like to notice. If the process is that of refrigeration, as referred to by Dr. Arnold, we will do well to remember the tooth cannot be subjected to great change of temperature without disturbance in the pulp. If the pulp had been subject to irritation in any degree, before we began to operate, then repeated shocks given in the manner described would most likely convey it past the point, when it would recover its physiological state. I can hardly believe that any benefits from the use of chloroform or ether in this manner are due to their properties as anæthetics. If heat is used no matter how, we affect a change in the organic tissues of dentine. It is changed physically, and fails to respond to injury. In these discussions we are sometimes led to believe we are treating only hyper-sensitive dentine. If this was true, the rational treatment would be to exclude irritants and give the tissues rest. Unfortunately healthy dentine is frequently exquisitely sensitive. The obtunding agents to meet all these conditions have yet to be discovered.

*Dr. Taft*—I think the treatment as indicated by Dr. Custer will be liable to do injury in the direction indicated. It is too energetic and there might be liability of producing periosteal irritation. There would be very considerable liability of injuring the pulp of the tooth to an extent that it would not recover upon the return of the normal temperature. Now, some very broad claims are made for the use of heat alone, it is a reasonable method of treatment; a treatment that in most instances perhaps will be effectual, in all instances if pursued to a sufficient extent. Frequent application of headed air during excavating will usually make the operation quite bearable by almost every patient. The use of an ether spray alone is of very little value in ordinary cases.

After the excavation has been completed and thorough desiccation, then a solution of cocaine or heated carbolic acid may be used with decidedly good effect. After excavation and desiccation a solution or varnish of gum mastic or copal may be applied to the walls of the cavity for the purpose of preventing a recurrence of the sensitiveness after the filling has been completed. By this means, in most cases, entire freedom from a return of the sensitivenes is secured.

*Dr. Arnold*—I do not wish to be understood as having used this remedy and I have never advocated it. It has been very unsatisfactory with dentists generally, sometimes I use chloroform and ether. Quite often very nervous patients, hysterical ones especially, have to be treated very carefully and cautiously. There is a question in my mind, whether by extreme temperature we do not impair the tissue to that extent that there would be trouble to the pulp afterwards.

*Dr. Bell*—Sensitiveness of dentine is a subject that is of the utmost importance to us. Many of us have used with great success alcohol upon dentine. This article is of great assistance in our practice, but I make application of this in a different way from the ordinary manner in which it is used. I apply with cotton and then use the hot spray. Make an application of peppermint, and if it is an extreme case apply a hot blast two or three times, as the case may require, until all of the moisture has been removed. I use the bur, drill, or excavator; in this way I can excavate much more rapidly and much more effectually. The bur, or drill, or excavator frequently becomes hot and produces pain in that way. I agree that it is always policy to use some non-conductor in the method of filling teeth.

*Dr. Wright*—The question at issue is a very difficult one, about which to get reliable data. What would be a success in the hands of Dr. Custer may not be in my hands. We have been talking about dentine and anæsthetic agents ever since I can remember, and that is a very long time ago, and it seems to me with all our desire and determination we will be able to get something that will reduce pain. We have not gotten any nearer to it than we were twenty-five years ago. The pain is, of course, in a nerve center, and when we have difficult and serious operations to perform, it seems to me, that we may do as the surgeon does and rely upon such anæsthetics as chloroform, ether, etc. We cannot get reliable data. Only a few years ago we were all interested in the question of using hot glycerine and carbolic acid, which had such a bad smell and disturbed the patient and gave an offensive odor to the operating room, but for the present we have not a reliable local anæsthetic that will do away with this pain.

*Dr. Wilson*—I rise simply to call attention to this matter in the way of caution, and as you all seem to agree that we have nothing that is perfect, the caution is, that in many respects we are producing more pain than we are relieving, in the use of hot air or warm air, if it is not applied carefully. We have never been able to wholly control this affection, and I think that we all agree that hot air when applied carefully gives some relief, but we must use it cautiously.

*Dr. Smith*—We must remember that we work and cut upon a tissue unlike any other in the human body. It is the question of the character and nature of the affection which has not been settled. It has been the general opinion that the injury is first upon the dentinal fibre itself. It may, however, be directly upon the pulp. If this is true, we may not expect much benefit from our treatment with obtundents.

It is very difficult for a patient to realize when you touch the dentine, that the hurt is not right at the surface, but it don't necessarily follow that it is. And I think Dr. Wright is correct in what he proposes, that we must give an anæsthetic, that we must put the nervous system to sleep before we can treat the patient.

*Dr. Wright*—Dr. Smith refers to the tissue being different from any other, and this is why we cannot apply local anæsthetics. We can apply the fumes of chloroform to a wound and in that way reduce, to a certain extent, the sensibility. I appreciate that the dentine and the enamel of teeth are different tissues, and still I hold that we are not any nearer to it than we were twenty-five years ago.

*Dr. Taft*—I can hardly agree with Dr. Wright, when he says that we have been working twenty-five years, and have not made any advancement on this subject; in other words, we are not any nearer the desired result than at that time. I can hardly understand why such statements should be made; my own experience will not allow unchallenged, a statement of that kind:

and coming as it does from a professional brother, I can hardly let it pass without some modification. I know, as I think every operator doeshere, that he manages his cases, better than he did twenty-five years ago—better than he did ten or fifteen years ago; we are making progress in this particular as well as in other things.

I have heard the same statement made in regard to other branches of our practice: "We are no better now than we were half a century ago." Now, everybody knows that this is not true. Men making such statements may sometimes persuade themselves that they are true.

In this particular matter you do not need air as hot as it can be made; the temperature can be regulated according to the case. It is simply a current of warm air to facilitate the removal of the moisture from the cavity; and it is a mistake whenever pain is produced by the introduction of a current of air into the cavity. The air should be so regulated in temperature, and in the rapidity of the current, that little or no pain is experienced by the patient; and it is an unskillful operator who does otherwise.

Prof. Guilford, of Philadelphia, has been giving special attention to the use of warm air for this purpose, and to apparatus best adapted. A full description of Dr. Guilford's device is given in the Transactions of the Ninth International Medical Congress, vol. 5, page 472.

The temperature can be regulated by adjusting the point of the air-pipe at a proper distance from the tooth, or by having the air in the instrument of the right temperature. With the proper appliances, the temperature can be perfectly controlled. In the use of the ether spray the temperature is reduced and in this way the sensitiveness is lessened.

*Dr. Robinson*—Dr. Taft was speaking about the practice of drying a cavity, and I will speak of a very inexpensive apparatus that works well. I have a little globe cylinder, and on each side there is a little point running out, to which I attach a little rubber tube; on one end is attached a rubber ball, and on the other is a little mouth-piece—something like that with a very fine blow-pipe jet. I let it get very hot and use the bulb with one hand and place the little tube over the cavity with the other; and I can get any desired heat I wish. I like it better than any other apparatus that I have ever used for that purpose. As to sensitive dentine that Dr. Wright was speaking about, I remember when we were students at the Ohio College there was a great deal said upon the subject of sensitive dentine, and it was one of our particular topics in the class; and we did not know how to get rid of it. And always since then I have more or less taken a great deal of pains to find a plan of operating to save my patients as much pain as possible; and I have found nothing to-day of any consequence except to warm the cavity as much as I think it ought to be without producing pain, and then to excavate a little while, and drying it again, and keep on in that manner until I get through with the operation as well as I can and possibly better than with any other method I have ever used.

There is a condition in which were we to use a dry cavity, there is more sensitiveness than when there is moisture; and it requires a great deal of judgment in these cases to know when to dry a cavity and when to let it remain moist. There are many cases in which we go to work without applying the dam at all. Preparatory to putting on the dam, we operate and have the cavity pretty well prepared. We put on the dam, for instance, and find out that it is almost impossible to excavate the cavity at all on account of its extreme sensitiveness. With moisture you can get right along and excavate the cavity without any pain to the patient at all, until in gets dry again; and then the reverse may occur. Where we treat the cavity with no moisture at all in it, you can hardly excavate it to the slightest degree. The only question for us is to decide how best to excavate a cavity and with as little pain as possible.

